# Squamous Cell Carcinoma Developing in a Buccal Mucosa Graft after Urethroplasty: A Report of 2 Cases of Malignant Degeneration

**DOI:** 10.1155/2021/5569373

**Published:** 2021-07-24

**Authors:** Catti Massimo, Nappo Gerocarni Simona, Tadini Barbara, Cerchia Elisa, Ferrero Luisa, Gambella Alessandro, Pacchioni Donatella, Elisabetta Teruzzi, Marco Falcone, Sedigh Omidreza, Gontero Paolo

**Affiliations:** ^1^Paediatric Urology, Città Della Salute e Della Scienza Torino, Italy; ^2^Pathology Unit, Department of Medical Sciences, Città Della Salute e Della Scienza Torino, Italy; ^3^Pathology Unit, Città Della Salute e Della Scienza Torino, Italy; ^4^Urology Department, Città Della Salute e Della Scienza Torino, Italy

## Abstract

Buccal mucosa graft (BMG) was originally described in 1992 for the treatment of challenging cases of hypospadias (proximal or redo cases) and has gained increasingly popularity also when dealing with complicated urethral stenosis, as it is associated with a good outcome. The development of a malignancy in a BMG urethroplasty was reported for the first time in 2017. We report two more cases of a malignant degeneration of a BMG used in a urethroplasty to treat recurrent urethral stricture.

## 1. Introduction

AUA guidelines for the management of male urethral strictures include urethral dilation, direct visual internal urethrotomy, and urethroplasty. According to AUA and SIU recommendations, urethroplasty is the gold standard to treat long or recurrent urethral strictures. BMG, since its original description in 1992 [1], is considered the graft of choice in urethral surgery when dealing with challenging hypospadias and urethral strictures, as it provides good results. Only one case of tumor developing in a BMG urethroplasty was previously reported [2]; we describe the 2^nd^ and 3^rd^ cases.

## 2. Case Presentation

**Figure 1:**
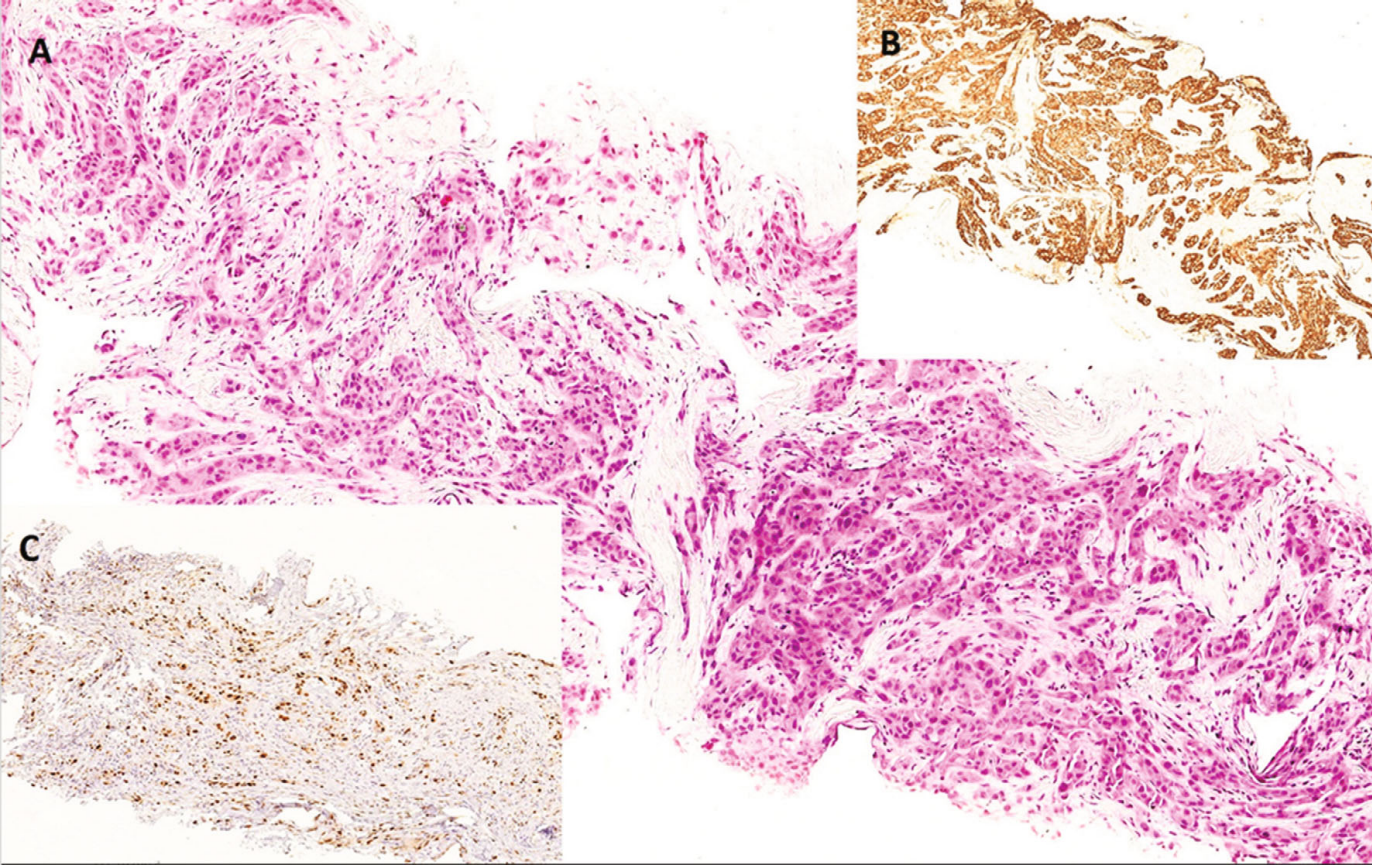
Diagnostic biopsy in case 1 (a) staining with hematoxylin and eosin; the epithelial origin is confirmed by the positivity to high MW cytokeratin (b); the proliferative index, evaluated with the MIB-1 clone of the Ki-67 antibody, highlights the malignant nature of the lesion with high proliferation (c).

**Figure 2:**
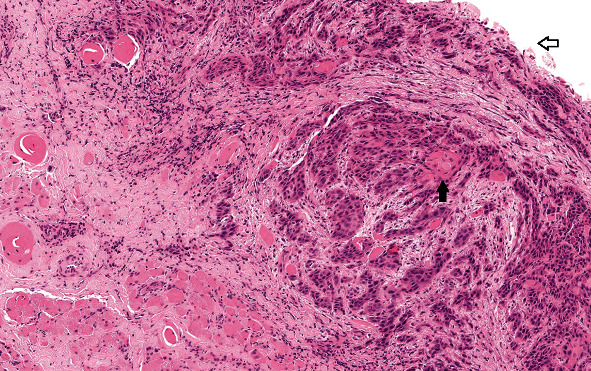
Representative sections of the surgical specimen of case 1 (surgical biopsy of bulbar urethra). Well-differentiated squamous cell carcinoma with a horn-like appearance of pearls (full arrow) and surface erosion (empty arrow). The lesion invades the dermis and muscle tissue (staining with hematoxylin and eosin).

**Figure 3:**
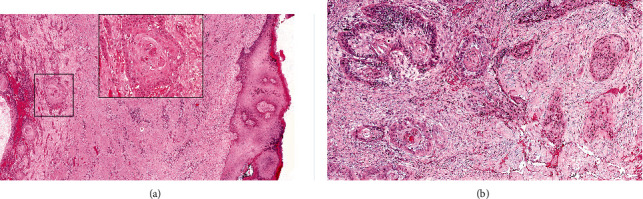
Representative sections of the surgical specimen of case 2 (hematoxylin and eosin staining): surgical resection specimen of the buccal patch (a; original magnification ×50); keratinized epithelium on the right side of the image on the left side lies the infiltrating carcinoma (inset: detail at higher magnification of squamous nature consisting in keratin pearl). Aggregates and nests of intensely eosinophilic neoplastic cells with densely, hyperchromatic nuclei and keratinizing features are widely identified all over the specimen, confirming the carcinoma's squamous nature (b; original magnification ×100).

1. A male smoker patient developed a postinfectious HPV-related bulbar urethral stricture which was unsuccessfully treated with 2 internal urethrotomies. He underwent ventral urethroplasty with BMG in 2008, at the age of 46 years. In 2011, he developed an HPV-related squamous papilloma of the tongue and genital condyloma. In 2013, he presented urethral discharge with dysuria and developed a bulbar nodule and inguinal adenopathy. A nodular, hard, nonstenotic lesion was detected at cystoscopy on the site of previous BMG implantation (bulbar urethra) and biopsied, showing poorly differentiated SCC (Figure 1). Normal buccal mucosa was found at oral exploration. Considering the presence of inguinal nodal diffusion and the absence of urethral stricture, chemotherapy with paclitaxel, cisplatin, and ifosfamide was completed. In 2014, obturator-iliac laparoscopic lymphadenectomy was performed for locoregional diffusion together with a new biopsy of the persisting bulbar lesion. Histology confirmed poorly differentiated SCC pT2N2 in the bulbar urethra (Figure 2). Despite new chemotherapy treatment with docetaxel and cetuximab, the disease rapidly progressed to metastatic diffusion and the patient passed away
2. A male smoker patient with a posttraumatic stricture of the bulbar urethra, after unsuccessful internal urethrotomies, underwent ventral BMG urethroplasty at the age of 45 years. After 6 months, he presented with hematuria, inguinal adenopathy, and a hard, nodular mass of the posterior urethra; endoscopy showed thickening of the perineal and bulbar urethra and histology showed infiltrating, poorly differentiated keratinizing SCC. Biopsy of the ulcerated oral site of BMG harvest showed hyperplasia and fibrosis of the chorion with no malignancy. The patient then developed major psychiatric disorders with suicidal and uxoricidal tendencies, refused the proposed radical surgery, and underwent excision of the perineal, bulbar, and penile urethra with suprapubic cystostomy. Histology showed poorly differentiated SCC of the urethra, diffusely infiltrating the corpora cavernosa (Figure 3). The patient refused any other treatment including chemotherapy and eventually died

## 3. Discussion

Primitive tumor of the urethra is rare, accounting for 1% of urinary malignancies [3]. It affects mainly males, with a reported incidence of 4.3 males and 1.5 females per million. According to the literature [3], the most frequent histological type is urothelial carcinoma (54-65% of the cases), followed by squamous cell carcinoma (16-22%). Major risk factors are represented by urethral stenosis, chronic irritation after intermittent catheterization or urethroplasty, external beam irradiation therapy, radioactive seed implantation, chronic urethral inflammation or urethritis following sexually transmitted diseases (STD) (i.e. condylomata associated with human papillomavirus 16), and lichen sclerosus [3].

Diagnosis is made in most cases by endoscopic biopsy in patients presenting with urethral bleeding and discharge, hematuria, obstructive, and irritative urinary symptoms. Treatment is multidisciplinary, including surgery, chemo, and radiotherapy, and is not standardized [4], especially in advanced disease. It is aimed at preventing and stopping local and regional lymphatic diffusion, since the prognosis is mainly influenced by nodal stage [3].

Tumors of the oral cavity account for 2% of all malignancies, with a male/female ratio of 2/1. In most cases (90%), histotype is SCC^5^. Major risk factors are smoking, alcohol assumption, and HPV-related oral infection [5].

We speculated about the origin of the urethral tumor in these 2 patients, debating whether they were primitive tumors of the bulbar urethra or malignant transformations of the implanted BMG. Furthermore, we focused our attention on trying to determine whether the tumors originally developed in the buccal cavity or if the oral mucosa grafts underwent malignant transformation after implantation in the urethra.

Consider that

1. the histological type was SCC, being the most frequent oral malignancy and less frequently insurging in the urethra
2. the tumors developed in the bulbar urethra, precisely at the site of implantation of the BMG
3. these patients presented with typical risk factors for oral SCC, such as smoking, and, in case 1, confirmed oral HPV+ papilloma
4. both patients had no evidence of other SCC localization in the oral cavity

We came to the conclusion that, most probably, these tumors originated from the implanted BMG as a result of these main oncological predisposing factors: chronic inflammation after urethroplasty and progression of chronical HPV infection in case 1.

This report underlines the importance of extensive endoscopic and histological evaluation of all urinary symptoms presenting in patients after urethral reconstruction, to early detect complications and the development of malignancy.

Furthermore, these reports of malignant transformation challenge the assumption that BMG urethroplasty is a totally safe procedure. In case 1, sexually transmitted HPV infection most probably played a crucial role in oncological transformation, and for this reason, it is mandatory to inform the patient about STD-related risks and to prevent HPV or other sexually transmitted infections.

## 4. Conclusion

We report the second and third cases of malignancy developing in a BMG used for urethroplasty. BMG is largely used in children and adults, when a challenging urethral surgery is needed for long or persistent urethral strictures or for proximal/redo hypospadias. This complication, although very rare, should be kept in mind when informing the patient about the potential risks of the procedure and when obtaining informed consent. Prevention of STD is mandatory in these patients, as it associates with an augmented oncological risk.

## Abbreviations

BMG: Buccal mucosa graft
SCC: Squamous cell carcinoma
STD: Sexually transmitted disease.
